# Supplementary material for: Causal Relationship between Aging and Anorexia Nervosa: A White-Matter-Microstructure-Mediated Mendelian Randomization Analysis
Source: Biomedicines. 2024 Aug 16;12(8):1874. doi: 10.3390/biomedicines12081874 (PMC11351342; doi:10.3390/biomedicines12081874)
Supplement: Supplementary file 1 [file biomedicines-12-01874-s001.zip › Supplementary Figure/Figure S8/Leaveoneout plot of MR analysis for TL to ubm-b-1486.pdf]

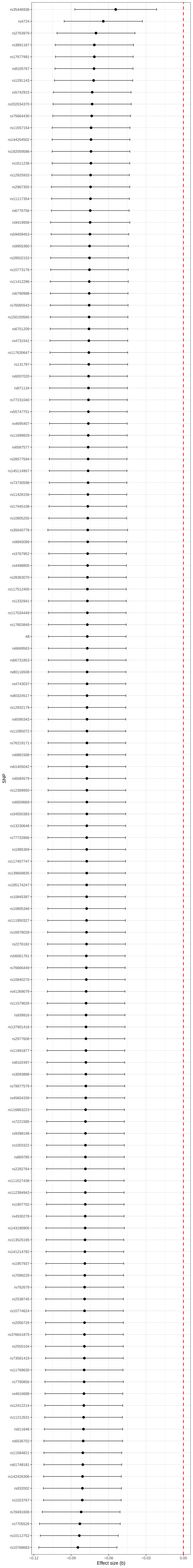
rs35446936
rs4724
rs2763979
rs3891167
rs17677991
rs8105767
rs1291143
rs5742915
rs202034370
rs75664430
rs11557154
rs144204502
rs182059586
rs1611236
rs12925933
rs2967355
rs11117354
rs6776756
rs9419958
rs59409453
rs9955360
rs28502153
rs10773176
rs11412296
rs6790988
rs76065543
rs150150565
rs6751209
rs4731541
rs117630647
rs131797
rs6007020
rs871134
rs77231040
rs55747751
rs4695407
rs11699829
rs6587577
rs28577594
rs145114957
rs73730598
rs11426156
rs17445108
rs10905255
rs35640778
rs9940099
rs3767952
rs4498805
rs28363070
rs117512405
rs1332941
rs117034449
rs17803849
All
rs6669563
rs66731853
rs80116508
rs4743037
rs80324517
rs12932179
rs6590343
rs11085072
rs76219171
rs6881568
rs61405042
rs6584579
rs12369950
rs6659669
rs34550383
rs13230646
rs77732866
rs1985369
rs117407747
rs139669835
rs185174247
rs10845387
rs10805346
rs111950327
rs16978028
rs2276182
rs56061761
rs76666449
rs10840270
rs41269079
rs11579626
rs939916
rs137901416
rs2977608
rs11991877
rs8102497
rs3093888
rs79977579
rs45604339
rs116863223
rs7221585
rs9398196
rs1003322
rs869785
rs2282764
rs111527438
rs112394943
rs1907702
rs4530278
rs143190905
rs113525195
rs141214782
rs1957937
rs7099229
rs762679
rs2538745
rs10774624
rs2056726
rs376641875
rs2555104
rs73581419
rs11769630
rs7790856
rs4616688
rs12412214
rs11212631
rs611646
rs6536702
rs11584821
rs61748181
rs142426306
rs932002
rs1023767
rs78491606
rs7705526
rs10112752
rs10768683
SNP
-0.12
-0.09
-0.06
-0.03
0.00
Effect size (b)
